# Reducing empiric piperacillin-tazobactam use for patients with community-acquired intra-abdominal infections

**DOI:** 10.1017/ash.2026.10391

**Published:** 2026-04-28

**Authors:** Claire Wilson, Kevin Epps, Courney Willis, Julio Mendez, Joseph Kim, Jesse St. Clair, J. Colt Cowdell

**Affiliations:** Mayo Clinic in Florida, USA

## Abstract

**Objectives:**

Community-acquired intra-abdominal infections (CA-IAIs) are a leading cause of US hospitalizations. Piperacillin-tazobactam is often used to empirically treat CA-IAIs, despite national guidelines recommending narrower-spectrum antibiotics for these infections. The overuse of broad-spectrum agents such as piperacillin-tazobactam contributes to antibiotic resistance, which poses serious public health challenges. This resident-led quality improvement initiative aimed to reduce unnecessary piperacillin-tazobactam use for treating CA-IAIs measured as DOT/1,000 patient-days by 10% without adversely affecting hospital length of stay (LOS).

**Methods:**

Using the DMAIC (define, measure, analyze, improve, control) framework, we identified barriers to appropriate antibiotic use and developed a treatment algorithm for CA-IAIs that included clear guidelines and exclusion criteria. This algorithm was disseminated to internal medicine residents and emergency department physicians along with educational sessions to highlight updated CA-IAI treatment recommendations, antibiotic resistance, and appropriate antibiotic ordering via the electronic health record. Antimicrobial stewardship pharmacists provided overnight support to assist with de-escalation. Data were collected over a 10-month period spanning 2 intervention phases. The primary outcome was piperacillin-tazobactam use, measured as days of therapy (DOT) per 1,000 patient-days and DOT per patient LOS. Mean LOS served as the balancing measure.

**Results:**

Piperacillin-tazobactam use was significantly reduced (*P* < .001) after the interventions without increasing the mean LOS.

**Conclusion:**

This project raised awareness of antibiotic resistance and led to lasting improvements in reducing the inappropriate use of piperacillin-tazobactam to treat CA-IAIs, without affecting the mean LOS. This was attributed to the strong collaboration among a multidisciplinary team of infectious disease physicians, antimicrobial stewardship team members, residents, emergency department physicians, and faculty.

## Introduction

Community-acquired intra-abdominal infections (CA-IAIs) are one of the leading causes of hospitalizations in the US annually and are a major cause of morbidity and mortality in hospitals worldwide.^1^ The cornerstones of treatment include timely and adequate source control with appropriate antimicrobial therapy.^2^ Piperacillin-tazobactam is a broad-spectrum antimicrobial commonly used to treat IAIs because it provides coverage against a variety of gram-negative bacteria (eg, *Escherichia coli* and *Pseudomonas* species), gram-positive bacteria (eg, *Streptococcus* species), and gastrointestinal tract anaerobes.^3^

A primary concern with the overuse of anti-pseudomonal regimens such as piperacillin-tazobactam to treat CA-IAIs is that overexposure may lead to selective pressure and the emergence of resistant *Pseudomonas* strains. The Centers for Disease Control and Prevention have classified multidrug-resistant *Pseudomonas aeruginosa* as a serious threat to public health, with an estimated 32,600 cases in hospitalized patients, 2,700 estimated deaths, and attributable public health care costs of $767 million in 2017.^4^ Recent data published by the Centers for Disease Control and Prevention revealed 6 hospital-acquired antimicrobial-resistant bacterial infections, including 1 case of multidrug-resistant *P aeruginosa*. These infections increased by 20% during the COVID-19 pandemic compared with prepandemic times, peaking in 2021 and remaining above prepandemic levels in 2022.^5^

In 2017, the Surgical Infection Society published guidelines recommending narrow-spectrum antimicrobial regimens for CA-IAIs.^6^ Despite these guidelines, a study by Lodise et al.^7^ showed that more than half of patients with low-risk CA-IAIs continued to receive piperacillin-tazobactam. In a study by the Infectious Disease Society of America, 77,663 patients with qualifying hospitalization and evidence of CA-IAI were retrospectively analyzed.^7^ These patients represented 2% of all admissions during the study time frame. Of the 46,722 patients with CA-IAI who met the criteria for low-risk infection, more than 75% of patients were treated empirically with fluoroquinolones or piperacillin-tazobactam outside of established guidelines. Notably, the rates of resistance to fluoroquinolones are also increasing, exceeding 25% in most regions in the US, which limits their utility as an effective first-line treatment.^8^ When treating CA-IAIs, avoiding unnecessary piperacillin-tazobactam use through antimicrobial stewardship endeavors is important for preventing drug resistance.

This quality improvement project aimed to decrease empiric piperacillin-tazobactam use for patients presenting with low-risk CA-IAIs without increasing hospital length of stay (LOS).

## Methods

### Study design

The preintervention period for this project was from September 1, 2023, to January 31, 2024, and was followed by 2 plan-do-study-act (PDSA) intervention cycles until the end of May 2024 (PDSA 1: February 2, 2024–March 31, 2024; PDSA 2: April 1, 2024–May 31, 2024). The project was completed over a period of 10 months. Our group followed the DMAIC (define, measure, analyze, improve, and control) method for quality improvement and compared data from before, during, and after the 2 intervention phases, described below. Data were collected and analyzed with Medication Management Analytics, which is integrated into the hospital’s electronic health record system. This program is part of the Mayo Clinic antimicrobial stewardship program’s Tableau dashboard (Tableau Software, LLC), which details antibiotic use data that can be extracted for requested periods. Patients were included who were admitted and treated by all 4 teams of internal medicine residents and prescribed piperacillin-tazobactam for an indication of CA-IAI including diverticulitis, appendicitis, infectious colitis, cholecystitis, hepatic abscess, and peritonitis at Mayo Clinic in Florida (See Figure 2). Exclusion criteria were patients who were over the age of 70 with history of malignancy or organ transplant, patients diagnosed with sepsis or septic shock, cholangitis, or pancreatitis, patients who received IV antibiotics for 90 day period, or patients with prior *Enterococcus* or *P. aeruginosa* isolated from intra-abdominal fluid or abscess cultures and/blood within the last year. The intervention targeted patients with presumed low-risk CA-IAI in whom guideline-recommended therapy would not routinely require empiric antipseudomonal coverage. Patient data were extracted for the period between September 1, 2023, and January 31, 2024.

### Interventions and time line

**Figure 1.**
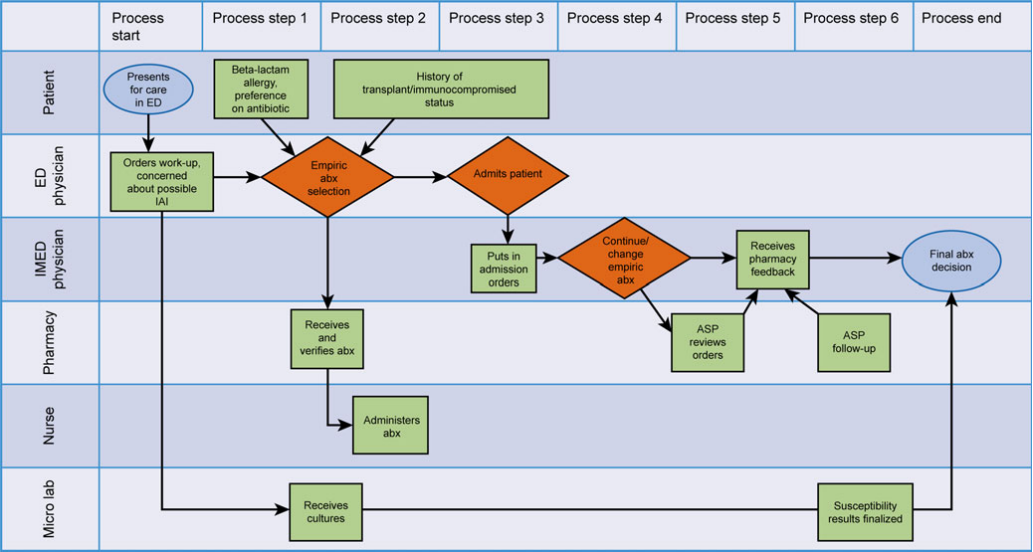
Swimlane diagram for process mapping. Abx indicates antibiotic; ASP, antimicrobial stewardship pharmacist; ED, emergency department; IMED, internal medicine; IAI, intra-abdominal infection; micro lab, microbiology laboratory.

We used a swimlane diagram to map out the processes contributing to piperacillin-tazobactam use (Figure 1). We also used a fishbone diagram and a Pareto chart to identify key causes of unnecessary piperacillin-tazobactam use. Key causes included prevalent hospital culture, perceived broad coverage of the antibiotic, and lack of knowledge regarding current best practice guidelines. Based on our key cause analysis, we designed and implemented the interventions described below. The choice of intervention pursued was ultimately decided on the basis of an impact effort grid.

#### PDSA1 (February 2, 2024-March 31, 2024)

**Figure 2.**
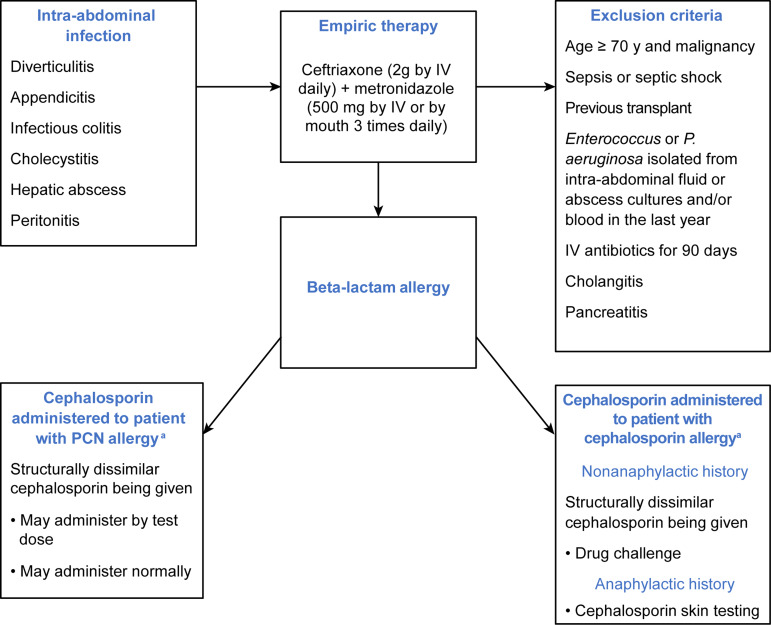
Algorithm of treatment guideline disseminated to resident teams. IV indicates intravenous; PCN, penicillin. ^a^exclude patients with penicillin or cephalosporin-related severe cutaneous adverse reactions, Stevens-johnson syndrome/toxic epidermal necrolysis, drug reaction with eosinophilia and systemic symptoms, acute generalized exanthematous pustulosis, and generalized bullous fixed drug eruptions. Used with permission of Mayo Foundation for Medical Education and Research.

We conducted a didactic session for residents on the importance of antibiotic resistance and the evidence-based management of CA-IAIs and held a separate meeting for emergency department physicians. We also designed an algorithm of treatment guidelines for patients with CA-IAIs (Figure 2) that was disseminated to residents and faculty. The algorithm included common infections, exclusion criteria, and steps for patients with documented beta-lactam allergy. The algorithm of treatment guidelines was posted in common workspace areas, included in weekly email updates, and shown during educational sessions.

#### PDSA2 (April 1, 2024-May 31, 2024)

We optimized off-hour education and conversation between physicians and pharmacists by recruiting a nighttime antimicrobial stewardship team of pharmacists. The pharmacists reviewed all orders for piperacillin-tazobactam and contacted the ordering physicians directly through secure messaging in the electronic health record with suggestions to de-escalate the antibiotic if criteria were met to do so. Provider acceptance of recommendations was encouraged through multidisciplinary discussions and repeated educational enforcement.

### Data collection and analysis

Postintervention data collection began 1 month after the first educational session and guideline dissemination, with 2 subsequent PDSA cycles implemented over the next 2 months. We compared preintervention and postintervention data to assess changes in piperacillin-tazobactam use, which was our primary outcome measure. Days of therapy (DOT)/1,000 patient-days was our selected metric for antibiotic consumption. According to the Infectious Diseases Society of America Antibiotic Stewardship guidelines, this is the preferred method and is an accepted alternative for measuring defined daily antibiotic doses. DOT was estimated by calculating the number of days a patient was prescribed each antimicrobial. The number of combined days was then normalized to the patient volume by dividing by 1,000 patient-days during the designated reporting period. After normalizing for patient volume, we could use DOT to compare antimicrobial use between institutions; however it should be emphasized that this study was conducted at a single academic medical center.

DOT/LOS per patient was used as a supplementary measurement for comprehensive statistical analysis. LOS was used as a balancing measure because our team wanted to ensure that the intervention did not lead to an increased duration of hospitalization by potentially undertreating CA-IAIs. The patients included in the balance measure were admitted to the residents’ service and prescribed piperacillin-tazobactam, ceftriaxone, metronidazole, ampicillin/sulbactam, or amoxicillin/clavulanate potassium for CA-IAIs. Statistical analyses were performed using GraphPad Prism version 10 and Tableau analytics tools integrated within the institutional antimicrobial stewardship dashboard. Continuous variables were compared across the preintervention and postintervention phases. Because the data did not meet assumptions of normal distribution and sample sizes were relatively small, nonparametric testing was used. The Kruskal-Wallis test was applied for comparisons among the three study periods, followed by Dunn multiple comparison testing to identify differences between groups. A *P* value of less than .05 was considered statistically significant.

## Results

**Figure 3.**
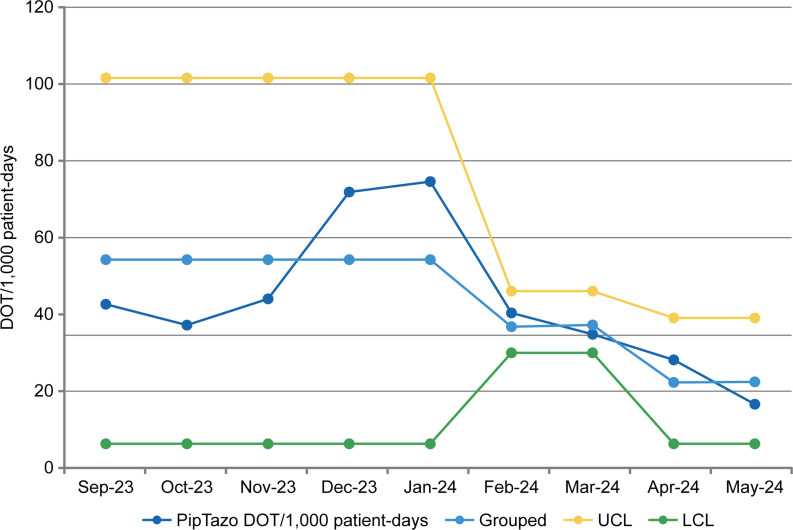
Piperacillin-tazobactam use for treating community-acquired intra-abdominal infections during the study period. For the line labeled “PipTazo DOT/1,000 patient-days,” each point represents the value for that month. The line labeled “Grouped” represents the mean values over the specified intervals. The change in control limits in February 2024 suggests a recalculated mean and limits due to a significant change in the data trend. DOT indicates days of therapy; LCL, lower confidence limit; PipTazo, piperacillin-tazobactam; UCL, upper confidence limit.

Data collected during the preintervention period revealed 73 patients received piperacillin-tazobactam for CA-IAI and a piperacillin-tazobactam use rate of 54.1 DOT/1,000 patient-days (Figure 3) and 0.3 DOT/LOS per patient. Mean LOS during this period was 7.8 days among the total 162 patients treated for CA-IAI. Our team aimed to decrease the DOT/1,000 patient-days by 10%, to 48.7, within 6 months (and DOT/LOS per patient to 0.2) without negatively affecting LOS.

After the first intervention, which involved resident and faculty education and algorithm dissemination, 18 patients received piperacillin-tazobactam and the piperacillin-tazobactam use rate was 36.9 DOT/1,000 patient-days (Figure 3), and the DOT/LOS per patient was 0.2. The mean LOS decreased to 5.4 days among the total of 59 patients treated for CA-IAI. After the second intervention, which involved the recruitment of a nighttime pharmacist team, 14 patients received piperacillin-tazobactam, and the piperacillin-tazobactam use rate was further decreased to 22.3 DOT/1,000 patient-days (Figure 3), and the DOT/LOS per patient improved to 0.1. Mean LOS was stable at 7.0 days among the total of 85 patients treated for CA-IAI.

**Figure 4.**
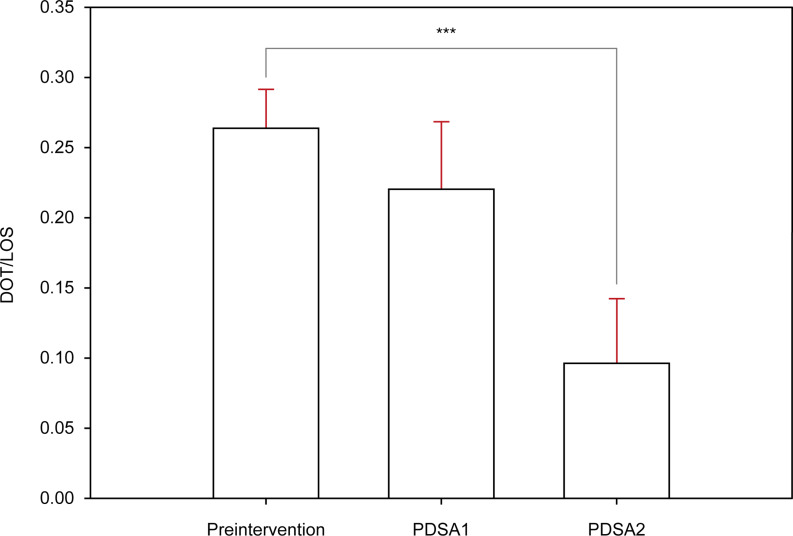
Significantly decreased piperacillin-tazobactam use after interventions. Values represent the mean DOT/LOS for each period. The red error bars represent the standard error of the mean. DOT indicates days of therapy; LOS, length of stay; PDSA, plan-do-study-act cycle. ***P < .001.

When preintervention data were compared with data after the first and second interventions, we found that the DOT/LOS per patient was significantly reduced after the second intervention (*P* < .001) (Figure 4). This reduction occurred after an improvement in antibiotic stewardship without adversely affecting patient LOS. Patient demographics and infection characteristics were similar across study periods, with most infections representing appendicitis, diverticulitis, or infectious colitis.

## Discussion

The primary goals of this resident-led quality improvement project were to reduce the unnecessary use of piperacillin-tazobactam in patients with CA-IAIs and to ultimately raise awareness of antibiotic resistance. Our efforts led to a reduction in piperacillin-tazobactam use, with rates decreasing from 54.1, to 36.9, to 22.3 DOT/1,000 patient-days, and from 0.3, to 0.2, to 0.1 DOT/LOS per patient, from the preintervention phase, to post PDSA1, to post PDSA2. The mean LOS decreased after the first intervention, from 7.8 days to 5.4 days, and remained stable after the second intervention at 7.0 days. This project represents an early implementation experience demonstrating the feasibility of reducing empiric antipseudomonal antibiotic use through resident-led antimicrobial stewardship interventions. Although the study period was relatively short, the observed reductions suggest that targeted educational and stewardship strategies can influence prescribing behavior in the inpatient setting. These findings align with those of other studies^1–7^ and support the use of more narrow-spectrum antibiotics for specific patient populations with a low risk of complications from infection. Analyzing cost implications in future initiatives would be a valuable addition to the literature.

One driver of broad-spectrum antibiotic use in low-risk CA-IAIs is concern for *Pseudomonas aeruginosa*. However, this risk appears to be overestimated. In a multicenter study, *P aeruginosa* was identified in fewer than 5% of culture-positive low-risk cases.^9^ Consistent with this, guideline recommendations do not support empiric antipseudomonal coverage in the absence of sepsis, hemodynamic instability, or other high-risk features, as it has not been shown to improve outcomes.^10^

These findings suggest that prescribing practices are influenced less by evidence and more by a culture of defensive or “just-in-case” medicine, in which clinicians favor broader coverage despite guideline recommendations. Prior work has similarly shown that clinicians often do not perceive broad-spectrum therapy as problematic.^7^ In our study, clinicians frequently believed that broader coverage was in the patient’s best interest, reinforcing this pattern.

Our findings are consistent with prior work demonstrating that clinicians may rely on “historical medicine” or anecdotal experience rather than current guidelines or local susceptibility data.^11^ Together, these data support the need for multifaceted interventions-including education, guideline dissemination, and clinical decision support-to promote more evidence-based antibiotic prescribing.

Initial antibiotic selection often occurs in the emergency department prior to transfer of care to the internal medicine team. In teaching hospitals, this decision is frequently made by trainees, and subsequent de-escalation may be limited by both inexperience and hierarchical dynamics, particularly when therapy was initiated by a more senior clinician. As a result, broad-spectrum antibiotics are often continued despite limited supporting evidence. These findings highlight the importance of targeted education for both emergency department and resident physicians, as well as multidisciplinary collaboration with antimicrobial stewardship pharmacists and infectious disease specialists to support evidence-based de-escalation.

When deciding which interventions to pursue, our team made an impact effort grid to discuss the various options. After seeing a partial improvement noted after PDSA1 and further discussion with our pharmacy team stakeholders, the team decided to move forward with the inclusion of a nighttime ASP team to review evening prescribing of all piperacillin-tazobactam orders, with specific targeted interventions for de-escalation in patients meeting criteria for CA-IAI. This nighttime ASP team significantly helped with the review of prescribing practices, which previously were only conducted during the daytime hours. Interventions considered but not ultimately pursued included adding a best practice advisory (BPA) into the electronic health record and modifying the electronic order set for piperacillin-tazobactam to guide prescribing practices. The impact of these interventions was perceived to be high, but the effort was also high because changing orders in the electronic health record system requires multicommittee approval and can take a prolonged time for approval. As this manuscript was written initially, this project was selected to be presented at the Mayo Clinic Quality Committee meeting, and further discussion is being had with leadership about the addition of a BPA to create lasting changes. We also considered creating a smart phrase for physicians to use in their documentation when ordering piperacillin-tazobactam and treating CA-IAI, with reminders on the latest guidelines. These interventions are still being considered for future use and will be reevaluated after the data are monitored by our antimicrobial stewardship team.

Our team learned several valuable lessons from this quality improvement project. First, recognizing potential barriers to intervention is important. Addressing common practices in hospital culture is difficult, and changing them is not easily done without open, extensive discussions about the reasoning behind clinical decision-making. One common challenge was resistance from physicians due to their comfort with using piperacillin-tazobactam for patients. They often wanted to wait for an extended period before de-escalating, despite the patient meeting criteria for lower-risk infection. This barrier was overcome with support from infectious disease colleagues and our antimicrobial stewardship team, who assisted with regular multidisciplinary meetings, educational sessions, regular updates communicated via email, and flyers posted in commonly occupied workspaces. Another barrier was time constraints for residents and faculty in scheduling sessions that allowed maximum participation and educational benefit. Our team benefited from maintaining monthly team meetings, allocating a faculty quality improvement sponsor, and designating one resident as team leader for seamless communication.

Although the primary focus of this project was antibiotic utilization, broader clinical outcomes such as mortality, infection recurrence, adequacy of source control, and readmissions were not systematically captured as part of this quality improvement initiative. Importantly, no concerning safety signals were observed during the intervention period, and the hospital LOS remained stable. Future studies incorporating additional clinical outcomes would further clarify the impact of stewardship-driven reductions in broad-spectrum antibiotic use.

The main limitations of this study include the fact it was conducted in a single academic medical center in a relatively short time frame of 10 months with modest sample size of patients. Because the intervention periods were relatively short, it is difficult to determine whether the observed changes represents sustained practice change or a temporary shift associated with increased awareness during the study period. In addition, the Hawthorne effect may have been introduced, given that resident physicians were aware that their orders were being monitored. Future research dedicated to replicating these findings in different settings and with longer follow-up periods would be of great value in assessing the sustainability of our findings.

### Conclusion

Antibiotic resistance, including multidrug-resistant *Pseudomonas*, is a growing worldwide public health concern that every health care professional must consider when treating patients with infection. Our findings show that narrow-spectrum antimicrobials adequately treat CA-IAIs without negatively affecting hospital LOS and support the use of guidelines provided by the Infectious Diseases Society of America and the Centers for Disease Control and Prevention when treating patients with CA-IAIs. In addition, our findings show the benefits of regular physician education and the value of antimicrobial stewardship teams in hospital settings.
